# Sensor Failure Detection in Ambient Assisted Living Using Association Rule Mining

**DOI:** 10.3390/s20236760

**Published:** 2020-11-26

**Authors:** Nancy E. ElHady, Stephan Jonas, Julien Provost, Veit Senner

**Affiliations:** 1 Department of Mechanical Engineering, Technical University of Munich, 85748 Garching, Germany; julien.provost@tum.de (J.P.); senner@tum.de (V.S.); 2 Department of Informatics, Technical University of Munich, 85748 Garching, Germany

**Keywords:** ambient assisted living, enhanced living environments, sensor failure, fault detection, fault isolation, smart home, non-intrusive sensors, binary sensors, event-driven sensors

## Abstract

Ambient Assisted Living (AAL) is becoming crucial to help governments face the consequences of the emerging ageing population. It aims to motivate independent living of older adults at their place of residence by monitoring their activities in an unobtrusive way. However, challenges are still faced to develop a practical AAL system. One of those challenges is detecting failures in non-intrusive sensors in the presence of the non-deterministic human behaviour. This paper proposes sensor failure detection and isolation system in the AAL environments equipped with event-driven, ambient binary sensors. Association Rule mining is used to extract fault-free correlations between sensors during the nominal behaviour of the resident. Pruning is then applied to obtain a non-redundant set of rules that captures the strongest correlations between sensors. The pruned rules are then monitored in real-time to update the health status of each sensor according to the satisfaction and/or unsatisfaction of rules. A sensor is flagged as faulty when its health status falls below a certain threshold. The results show that detection and isolation of sensors using the proposed method could be achieved using unlabelled datasets and without prior knowledge of the sensors’ topology.

## 1. Introduction

The ageing population phenomenon is one of the toughest challenges of this century. In 2019, 1 in 11 people around the globe was over 65 years old. This number of aged people is expected to rise to 1 in 6 people by 2050. The old-age dependency ratio is the ratio of the people over 65 to people between 20 and 64 years old. Some regions will witness this demographic shift the most, e.g., Europe and North America, will have an old-age dependency ratio of 49 per 100 by 2050 [1]. This demographic shift will induce challenges to governments as well as individuals [2]. The increasing ratio of retired persons to workers requires increasing the capacity of the social system. Moreover, as people grow into older age the chances of having age-related impairments and diseases increase, which if not monitored closely could lead to much worse health complications. Thus, the health-care costs are expected to increase as the population ages as well as the need for more care-givers. Stress would also be imposed on informal caregivers, e.g., family members. In order to decrease the burden on governments and individuals, promoting healthy ageing and independent living is becoming a priority. Exploiting the vast development of the information and communication technologies (ICT) and the emergence of ambient intelligence (AmI) is the key to providing such independence to older adults.

As a result, there has been an increasing interest in establishing Ambient Assisted Living (AAL) environments [2]. One of the definitions proposed for Ambient Assisted Living is “the use of information and communication technologies (ICT) in a person’s daily living and working environment to enable them to stay active longer, remain socially connected and live independently into old age” [3]. It is a multidisciplinary field that involves information and communication technologies, sociological sciences and medical research [4]. The AAL tools could be mainly categorised into health and activity monitoring tools, wandering prevention tools and cognitive orthotics tools [2]. The health and activity monitoring tools aim to monitor the activities of daily living (ADL) in an unobtrusive way, either to ensure the safety of the monitored person, or the completion of his activities, or to detect the deterioration in his cognitive and physical abilities. Wandering prevention tools were developed mainly to aid people suffering from dementia, while cognitive orthotics tools are used to aid people with cognitive decline. The AAL tools would cast some burden away from the family members of the older adults, decrease the need for qualified caregivers and have a positive impact on the psychological status of older adults as they would live independently at their homes for longer and more safely. To achieve the goals of the AAL systems, the following requirements need to be fulfilled; adaptability, interoperability, acceptability, usability and dependability [4].

Health or mobility related sensors are widely used for the monitoring purposes and represent the heart of the AAL environments [4]. Most of the sensors that are used for monitoring are event-driven binary sensors, for example the PIR sensor produces high output when motion is detected, otherwise it produces low output. Such sensors provide low level information, unlike the sophisticated information from cameras or microphones, and thus is more difficult to interpret and more prone to errors [5]. The failures that are encountered in such sensors are either fail-stop failures, where the sensor stops reporting values, or non-fail-stop failures, where the sensor reports values that do not reflect the occurring *events* that were supposed to be captured by it. Examples of the reported non-fail-stop failures that occur in AAL environments include sensors that get blocked by furniture, get remounted by the user in wrong locations, get stuck at a value or get spurious signals due to air drafts, sunlight rays or pets [6,7]. The traditional fault diagnosis methods for wireless sensor networks [8,9,10] are designed to deal with homogeneous, time-driven and continuous-valued sensors. However, such methods do not suit the nature of sensors installed in non-intrusive AAL environments, which are often heterogeneous, event-driven and binary sensors. This work aims to propose a sensor failure detection and isolation system for AAL environments equipped with event-driven, ambient binary sensors.

## 2. Related Work

A comprehensive literature review was presented by the authors of this article in [11], which focuses on the works concerned with detecting sensor failures, as well as tolerating its resulted faults, in AAL environments equipped with binary, event-driven sensors. The surveyed fault-tolerant systems focus mainly on location tracking [7,12,13] and activity recognition [6,14,15]. The sensor failure detection systems found in literature may be classified as model-based and correlation-based approaches [11]. The model-based techniques rely on deducing the location of the resident using the triggered sensors due to his movement or his performed activities. Then, this deduced location is compared with the location predicted either by his model of mobility, e.g., in [16,17] or by a localisation system, e.g., in [18,19]. The proposed model-based sensor failure detection approaches are not promising as they either use unrealistic models of resident motion that do not take into consideration previous locations and speed or install extra hardware that increases cost as well as the chances of errors. Fault detection and diagnosis frameworks that rely on modelling the sensors’ and actuators’ activation due to various user scenarios were presented in [20,21,22]. However, it can only detect failures in sensors that are involved in tasks that have sensor-actuator feedback.

The surveyed correlation-based techniques can be classified as methods based on exploiting sensor-appliance correlations, sensor-activity correlations and sensor-sensor correlations [11]. FailureSense [23] monitors the interval between motion sensor triggers and electrical appliances. Sensor failure is flagged during run-time when the monitored interval deviates from the previously learnt patterns from training datasets. The drawback of this method is that the assumption that the resident has to be physically beside the appliance to turn it on does not always hold. Idea system [24] first extracts the sensors that are triggered with each activity of daily living using an activity labelled dataset. In order to detect sensor failures, activity recognition is done, and whenever an activity is recognised while one of its sensors did not trigger, a rarity score is computed. Sensor failure alert is raised when the rarity score falls below a set threshold. The limitation of this approach is that it assumes that the activity has been correctly recognised in the first place. In addition, it requires labelled datasets for training. Following are the works based on the sensor-sensor correlations techniques. An approach based on temporal correlation and nonlinear time series analysis was investigated by Ye, Stevenson and Dobson; however, the experimental data was not enough to prove the effectiveness of this approach [25]. Same authors have proposed the use of density based clustering to detect outlier sensor triggers [26,27]. However, clustering occurs as a postprocess step on the collected data. SMART system uses simultaneous multiple classifiers, a classifier for each sensor failure. It detects a sensor failure by analysing the relative performance of these classifiers [6,28]. This approach lacks scalability and needs excessive training effort. DICE [29] extracts correlations and transitional probabilities among sensors and actuators offline. Failure is detected either when a sensor is missing from a predefined correlation or when a group of sensors fires despite having a zero transitional probability with the previous group of triggered sensors. The drawback of this approach is considering any group of triggered sensors as a correlation, even if it has only appeared once, thus questioning the reliability of correlations and making the approach more computationally complex especially when the number of installed sensors increases.

Our research work favoured adopting a correlation-based approach over a model-based approach, to avoid the disadvantages of relying on generic human mobility models, like in [16], that may not be accurate nor personalised to reflect the behaviour of the monitored person. In addition, adding extra hardware, as in [18,19], was avoided in order not to increase the implementation cost. Our proposed sensor failure detection and isolation system approach focused on sensor-sensor correlations rather than sensor-appliance and sensor-activity correlations. Sensor-appliance approaches [23] rely on assuming that there will be correlations between the activation of the electrical appliance and the triggering of the motion sensors in the areas leading to it, which is becoming less common in smart homes as most appliances can be switched on remotely. Meanwhile, failure detection using sensor-activity correlations [24] requires obtaining labelled data of performed activities to correlate the activities to the sensors during the training phase and relies on the accuracy of the activity recognition system at run-time to detect sensor failures. Our method does not need labelled datasets of sensor failures nor performed activities. It is based on extracting the nominal correlations between the installed sensors with no prior knowledge on the topology using unlabelled datasets. The association rule mining [30] technique is used to extract correlations. Unlike the approach presented in [29] that considers any proceeding triggers between sensors as a correlation, association rule mining extracts strong correlations that meet minimum relative support and confidence, which would ensure more reliability for failure detection. Association rule mining is characterised by its simplicity and good interpretability of results. There are works that have based their fault detection system on association rule mining; however, they were used to detect faults in time-series, continuous-valued data, e.g., [31,32]. Association rule mining has also been used for fault diagnosis using datasets that are already labelled with various system faults to associate which sensor signal values are responsible for corresponding system faults, e.g., [33]. In this paper, we propose a failure detection and isolation system for binary, event-driven sensors that is based on association rule mining. Association rule mining is refined to better suit our application. Postpruning is applied to get the most interesting correlations that the sensor failure detection and isolation system can rely on. The extracted correlations appear as a set of IF-THEN rules that indicate the sensors that trigger within a few seconds from each other. At run-time the set of rules are monitored and then the health status of each sensor is updated according to the satisfaction/unsatisfaction of the correlations. A sensor is flagged as faulty when its health status falls below a predefined threshold. Guidelines for the selection of the values of the parameters of association rule mining algorithm and the health status threshold are presented in Section 4.3.2. Failure detection and isolation take place at run-time; this is contrary to the approach in [26,27] that detects failure in precollected data. The approach presented in this paper is scalable; therefore, it overcomes this shortcoming found in the SMART system [6,28] which needs a large training effort to train a classifier for each sensor failure.

## 3. Sensor Failure Detection and Isolation System

**Figure 1:**
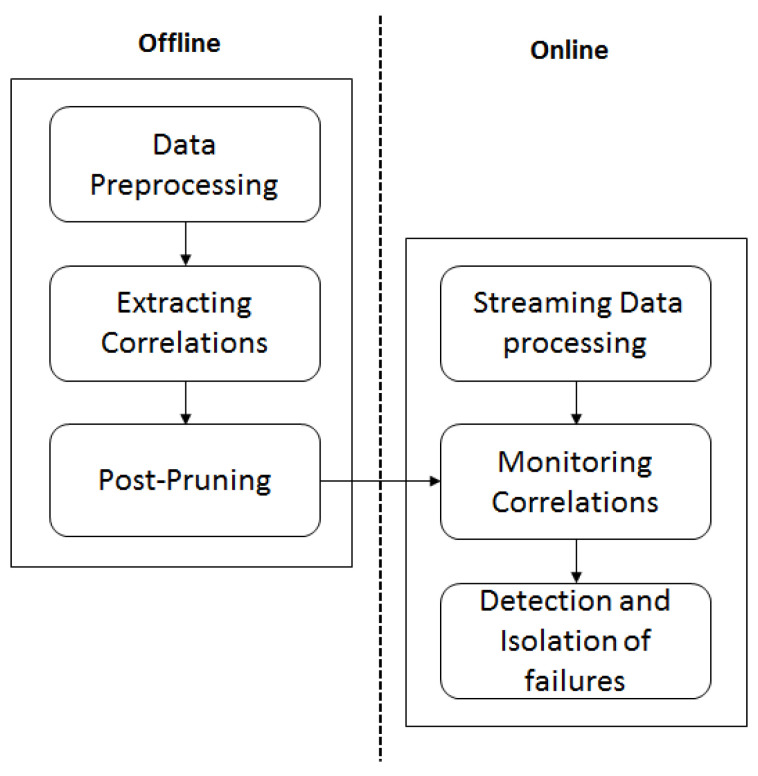
An overview of the proposed system.

Our sensor failure detection and isolation system consists of two stages: an offline stage and an online stage. During the offline stage, the fault-free sensor correlations are extracted from previously collected sensor dataset at the resident’s home during his nominal behaviour. Meanwhile online, the fulfilment of correlations are checked as sensor *events* are triggered by the resident and accordingly failure of sensors is determined. An overview of the proposed system is shown in Figure 1.

### 3.1. Sensor Correlations Extraction

First, preprocessing of training data is done, followed by rules extraction using association rule mining. Afterwards, the extracted rules are further pruned to obtain the most interesting sensor correlations.

#### 3.1.1. Data Preprocessing

The log obtained from AAL environments equipped with non-intrusive sensors consists of a series of *events*. Each *event* has a time stamp, sensor ID and the corresponding sensor *event* trigger. An example of a sensor *event* is 13 January 2011 10:28:14.65 M030 ON, which implies that sensor M030 has been positively triggered at the given time stamp. In order to extract correlations using association rule mining, the transformation of the time-stamped sensor *event* triggers dataset into a set of transactions takes place over a couple of steps. The first step consists of creating a multivariate time-series, where the value of each sensor is logged at every time stamp of the dataset in a separate sensor signal variable. Formally, let $s_{i,t}\in \left\{0,1\right\}$ be the value of the *i*-th sensor at timestamp t∈T. The set *T* is the set of timestamps of the log. For *n* sensors, concatenation produces the multivariate time-series *S*.

(1) $$S={\left\{\left(s_{1,t},s_{2,t},\ldots ,s_{n,t}\right)\right\}}_{t\in T}$$

Next, removal of all-zero rows is done. Formally, it corresponds to removing all-zero row vectors from the time-series *S*.

(2) $$V:=S\backslash {\left\{\left(0_{1,t},0_{2,t},\ldots ,0_{n,t}\right)\right\}}_{t\in T}$$

**Figure 2:**
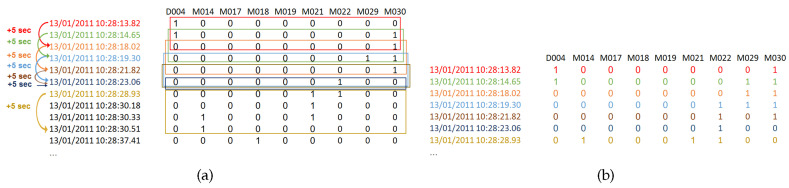
(**a**) Sliding window of size w= 5 s, is run over the multivariate time-series data. (**b**) Transactional database.

Figure 2a shows an example for a multivariate time-series created from an AAL log. At each row, a sliding window is used to group the sensors that have a signal value of 1 within the size *w* seconds of the sliding window via logical ORing. The output of the window will be a single transaction that has the time stamp of the start of the window. Formally, the value of the *i*-th sensor in the transaction computes to:

(3) $$d_{i,t}=sgn\left(\sum_{j\in [t,t+w]}v_{i,j}\right)$$

The sliding window is run over the multivariate time-series data to output a transactional database as illustrated in Figure 2, where each transaction presents the sensors that appear to be ON within *w* seconds from each other. The obtained sensors transactional database will be used in the upcoming correlations extraction step.

#### 3.1.2. Extracting Correlations

Correlations between fault-free sensors are extracted using the association rule mining technique. It is a data mining technique that was introduced by Agrawal et al. [30] and is commonly used on large transactional databases to find correlations between its items. Its most famous application is the market basket analysis, where the transactions of a supermarket are analysed to find which items are usually bought together by customers. Similarly, we aim to detect which sensors are most likely to be simultaneously active implying strong correlations.

A formal representation of the association rule mining problem is as follows. Let $I=\{I_{1},I_{2},\ldots ,I_{m}\}$ be a set of binary features denoted as items. Let the dataset T consist of a set of transactions $T=\{T_{1},T_{2},\ldots ,T_{n}\}$, where each transaction is a binary vector of items, e.g., if transaction $T_{1}$ contains only two items $I_{1}$ and $I_{3}$, then $T_{1}$ will have $T_{1}\left[1\right]=1$, $T_{1}\left[3\right]=1$ and the rest of $T_{1}$ vector are zeros. An association rule has the form of X→Y, where the antecedent X⊂I, the consequent Y⊂I and X∩Y=ϕ. The confidence of a rule denotes how likely it is to find item(s) of *Y* when item(s) of *X* occur(s), while the support of a rule is how frequent items of *X* and *Y* appear together in the dataset. Support and confidence, defined by Equations (4) and (5) respectively, are the most commonly used evaluation metrics that assess how strong the association rule is. The Apriori algorithm [34] is used to extract the association rules from transactional datasets. Minimum values for support and confidence have to be satisfied to avoid extracting meaningless rules. These minimum values need to be set by the designer. Lift is a metric used to confirm the dependency between the rule’s antecedent and consequent as shown in Equation (6), a value of 1 indicates independency, while greater than 1 indicates dependency. The higher the lift value, the greater is the dependency.

(4) $$\mathrm{Sup}\left(X\to Y\right)=\frac{|\mathrm{Transactions}\;\mathrm{containing}\;X\&Y|}{\left|\mathrm{Transactions}\right|}=P\left(X\cap Y\right)$$

(5) $$\mathrm{Conf}\left(X\to Y\right)=\frac{|\mathrm{Transactions}\;\mathrm{containing}\;X\&Y|}{|\mathrm{Transactions}\;\mathrm{containing}\;X|}=P\left(Y|X\right)$$

(6) $$\mathrm{Lift}\left(X\to Y\right)=\frac{|\mathrm{Transactions}\;\mathrm{containing}\;X\&Y|}{|\mathrm{Transactions}\;\mathrm{containing}\;X|*|\mathrm{Transactions}\;\mathrm{containing}\;Y|}=\frac{P(X\cap Y)}{P(X)P(Y)}$$

In the market basket analysis application, the items are the supermarket products, e.g., butter, bread, and a transaction contains the items that have been simultaneously bought by a customer in this transaction. In our AAL application, the items of the transactional database are the sensors installed in the AAL environment. However, a transaction contains the sensors that are ON simultaneously in an instant of time, as well as those sensors that are ON within its sliding window of size *w* seconds. This is because we are concerned to capture the temporal correlations between sensors within few seconds due to performing various activities by resident. The transactional database has been prepared in the preprocessing stage. Another concern in the AAL application is the uneven usage of the different areas of an apartment. A living room may be used by an older adult resident more often than the office room, leading to scarcity of the triggers of the office’s sensors in the dataset. In such cases, the support of the rule that has the less often triggered sensors may not exceed the minimum support value that was preset in the Apriori algorithm, and thus will not appear in the extracted set of rules. To overcome this limitation, we define a metric as relative support to be used in the Apriori algorithm instead of the support for rules extraction. Support compares the number of transactions containing all items of X & items of Y to the total number of transactions present in the database as shown in Equation (4). While relative support is defined by Equation (7), it compares the number of transactions containing all items of X & items of Y to the minimum number of transactions that contain any of the individual items of X or Y.

(7) $$\mathrm{Rel}.\;\mathrm{Sup}\left(X\to Y\right)=\frac{|\mathrm{Transactions}\;\mathrm{containing}\;X\&Y|}{\mathrm{Min}(|\mathrm{Transactions}\;\mathrm{for}\;\mathrm{each}\;\mathrm{item}\;\mathrm{in}\;X\;\mathrm{or}\;Y|)}$$

#### 3.1.3. Post-Pruning of Correlations

The mined set of rules that have already exceeded the minimum values for the relative support and confidence still needs further post-pruning to eliminate the redundant and/or less useful rules. Our proposed sensor failure detection method relies on the following hypothesis; if a rule has all of its antecedent sensors active during run-time, while its consequent sensors(s) did not become active within the specified sliding window size, then the sensors can be suspected to be faulty. Accordingly, we aim to have most of the sensors installed in the resident’s home appear in consequent part of rules so that they could be checked for being faulty in the monitoring stage. Hence, the rules are grouped for each sensor in consequent, i.e., if there are 20 sensors that appear in the consequent parts of rules, then we will have 20 groups. From each group, the rule with highest confidence, the rule with highest support and the two top trade-off rules between confidence and support, are selected. In our opinion, the former would be the most interesting rules to our application. To obtain the trade-off rules, confidence and support of the rules within each group are normalised, then are summed with weights 1:1, and the rules with the top two highest sums, i.e., trade-off scores, are selected. For example, to prune the rules of sensor M012, the rules that have M012 as a consequent are grouped, and then those rules which have the highest confidence, highest support and the two top trade-off scores are selected to be on the final set of rules that will be used in the monitoring stage, while the rest of the rules that have M012 as a consequent are eliminated.

### 3.2. Sensor Correlations Monitoring

The pruned set of rules are the most interesting correlations that will be monitored online; they are stored using bitmap arrays [35]. The health status of each sensor, which is the probability that a sensor is healthy, will be computed according to the fulfilment of these correlations.

**Figure 3:**
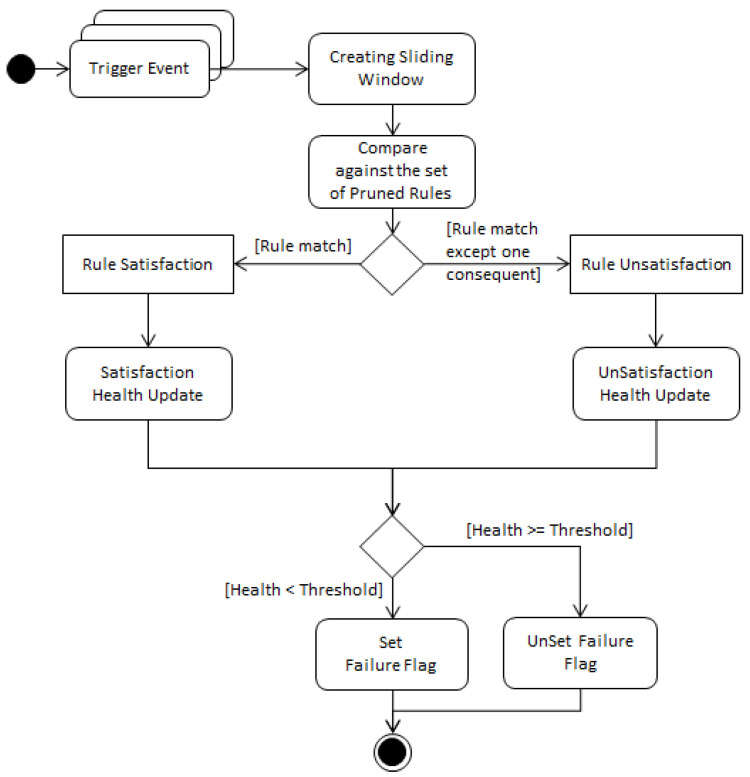
UML activity diagram of the health status update.

**Figure 4:**
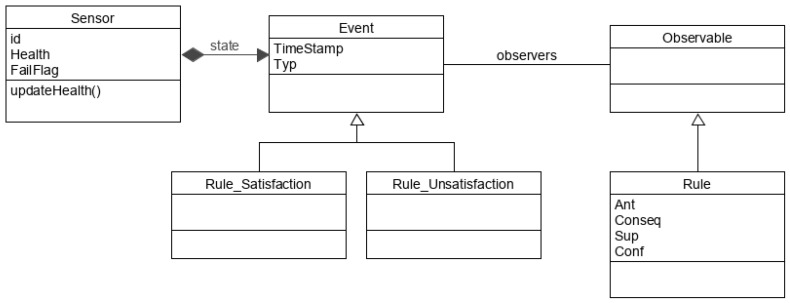
UML analysis object model of the online stage of the failure detection system.

Every time a sensor trigger *event* occurs, the data is processed and the corresponding sliding window is prepared similar to Section 3.1.1, where the sensor signal value is updated and the sliding window logically OR the sensors’ signals within the sliding window size of *w* seconds. A UML (Unified Modeling Language) diagram that describes the main workflow for the health status update is shown in Figure 3. The pseudocode in Algorithm A1 illustrates in details the health status update of sensors due to monitoring the pruned set of rules. Two satisfaction states of rules are possible: satisfaction and unsatisfaction. If the sliding window contains active sensors that satisfy a rule antecedent as well as its consequent, then this correlation is fully satisfied and the health status of these sensors are updated according to the satisfaction set of equations in Algorithm A2. It is assumed that only one sensor failure can occur at a time (single-sensor failure). Hence, if the sliding window contains active sensors that satisfy a rule antecedent but it fulfils the rule consequent except for one sensor, then this rule is unsatisfied. If this unsatisfied rule has already been satisfied in the previous sliding window or if it will be satisfied in the upcoming sliding window, then the health status will not be updated. In addition, if this rule has been unsatisfied in the previous sliding window then health will not be updated. Otherwise, the health status of this rule’s sensors are going to be updated according to the unsatisfaction set of equations in Algorithm A3. The joint probabilities between sensors that are included in the equations can already be obtained from the intermediate calculations of the Apriori algorithm while scanning the training data for finding the frequent itemsets, hence no extra computation is needed. Whenever the health status of a sensor falls below the preset health threshold, failure of this sensor will then be flagged. Figure 4 shows a UML analysis object model of the online stage of our system.

## 4. Experimental Work and Results

Our proposed approach for sensor failure detection and isolation was evaluated using a publicly available dataset. In this section, the methodology of the experimental work and the results will be presented.

### 4.1. Dataset

**Figure 5:**
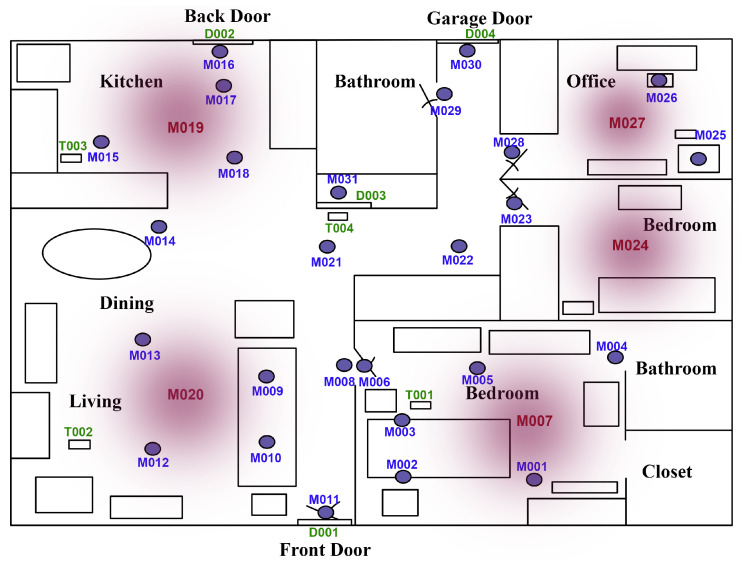
Aruba CASAS floor plan.

The publicly available Aruba CASAS dataset [36] was used to evaluate the proposed approach for failure detection and isolation of non-intrusive sensors installed in AAL. The dataset was collected over a duration of 6 months from a single-resident elderly’s home equipped with 31 motion sensors, 4 door contact sensors and 4 temperature sensors. As our approach is concerned with finding failure in event-driven binary sensors, temperature sensors were not included in the evaluation. In addition, the contact sensor D003, installed on a door located within the apartment as shown in Figure 5, does not have any triggers in the dataset. Thus in total, we have 34 sensors under investigation. The dataset was found to have some instances at which all of the sensors of the apartment get triggered at fractions of a second and all remain active for some time, thus filtering was done to remove such instances. To obtain the training and testing data, a split ratio of 50/50 was used. The training data was used for extracting the offline correlations, while the testing data was processed sequentially to simulate the run-time online processing using MATLAB 2019b software.

### 4.2. Evaluation Method

The following metrics are used for evaluating the sensor failure detection and isolation system: precision, recall and F1-measure. Precision is the percentage of true positives from the total number of sliding windows reported as positive, while recall is the percentage of true positives from the actual positive sliding windows. The testing dataset was divided into 6 segments, where the segment is approximately 2 weeks in length. Precision, recall and F1-measure are averaged over the segments.

In order to compute the true positives (TP) and false negatives (FN), the segments were duplicated and injected with failure. Failure is injected in each segment on each of the sensors that appear in the consequent parts of the extracted rules. Whenever a sliding window is reported to have a failure from our algorithm, the ground truth is compared with the report to determine whether it is a true positive or not. The start of sensor failure is chosen to be the first timestamp at which the sensor gets triggered in the segment. The faultless segments were used to count the false positives (FP) and true negatives (TN). Receiver Operating Characteristic (ROC) curve and the area under its curve (AUC) were also used to evaluate the performance of failure detection. The ROC curve shows the tradeoff between the true positive rate (TPR) and the false positive rate (FPR) as the health threshold value is varied from 0 to 1. The closer the curve to the left top corner of the plot, the better the performance of failure detection is, implying higher quality of rules that govern the failure detection. A diagonal ROC indicates that it is sort of random classification of failures.

### 4.3. Parameters of the Correlations Extraction

To achieve high performance for the sensor failure detection and isolation system, optimum values for four parameters need to be selected. These parameters are the sliding window size, minimum relative support, minimum confidence and health threshold. The optimum parameters would output the best set of correlations and thus the best failure detection and isolation performance. During the selection of parameters, thresholds setting dataset is used. The thresholds setting dataset contains 4-week data (2 segments) of the testing dataset.

#### 4.3.1. Parameter Effect

Before the selection phase, we wanted to study the effect of each parameter independently on the extracted rules and the performance of the system. Using the training dataset, we set the parameters and extract the correlations as described in Section 3.1. Then, the effect of the extracted rules on the performance of the failure detection system is evaluated on the threshold setting dataset that was injected with fail-stop failures. Fail-stop failure was injected for each of the sensors found in the consequent part of the extracted rules.

**Figure 6:**
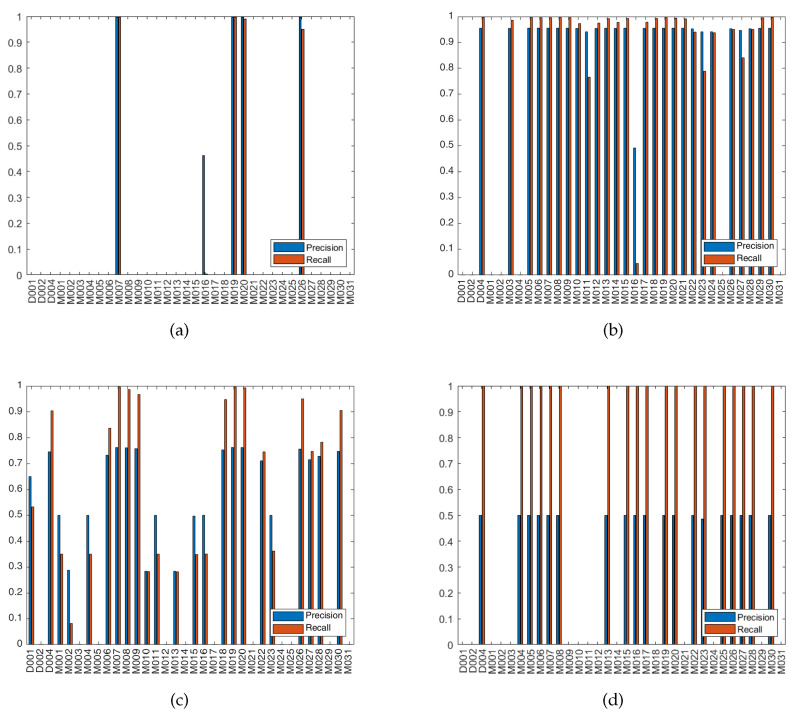
Precision and recall of failure detection when a sensor has fail-stop failure, at health threshold 0.4, (**a**) sliding window 0 s, minimum relative support 45%, and minimum confidence 60%. (**b**) sliding window 60 s, minimum relative support 45%, and minimum confidence 60%. (**c**) sliding window 0 s, minimum relative support 2%, and minimum confidence 60%. (**d**) sliding window 0 s, minimum relative support 45%, and minimum confidence 10%.

Increasing the size of the sliding window from 0 to 60 s, while keeping the minimum relative support at 45%, minimum confidence at 60% and health threshold at 0.4, was studied. It was observed that increasing the size of the sliding window increases the total number of sensors in the consequent parts of rules and increases the complexity of rules as well, i.e., more items/sensors per rule. Figure 6a,b plot the precision and recall of failure detection with the parameters set to the former values when the sensor ID of the x-axis is injected with fail-stop failure. For example, in Figure 6a the columns at sensor M007 show the values of precision and recall of failure detection when M007 was injected with fail-stop failure. High failure detection precision and recall can be observed in most of the cases of failed sensors. Note that the sensors with nonempty bar data in the figures are the consequent sensors of the extracted rules at the indicated values of parameters. Failure detection of only the consequent sensors were evaluated, i.e., in Figure 6a there are only 5 sensors that have bar data, denoting that only those sensors were present in the consequent parts of the rules extracted using 0 s sliding window, minimum support of 45% and minimum confidence of 60%, and failure was injected in each of those sensors and failure detection was evaluated then.

Figure 6a,c show the precision and recall of detecting failures with setting the minimum relative support at 45% and 2%, respectively, while maintaining the size of the sliding window at 0 s, minimum confidence at 60% and health threshold at 0.4. Observing the effect of decreasing the minimum relative support, it was found that the number of sensors in consequent part of rules increases but nearly half of them have low failure detection precision and recall. The low precision and recall are due to the low relative support of the rules that govern those sensors. Such sensors are the source of false positives, their governing rules seems to be spatially unrealistic, e.g., M001, M023 → M010, that was obtained using a sliding window of 0 s, implying that they are supposed to be ON simultaneously which cannot happen from a single resident even with the switch-off delays of motion sensors. The performance of the other sensors was also affected; the high false positives of the system have reduced their failure detection precision while maintaining their high recall. The complexity of the extracted rules has increased due to lowering the minimum relative support. Some sensors appeared in the consequent of rules when the sliding window has been increased but not when the relative support has been decreased, and vice versa. From Figure 6a–c, it is observed that D001, M001 and M002 have appeared in the consequent of rules, when relative support decreased from 45% to 2% and thus can be checked for being faulty, but they were not part of any rule’s consequent when the sliding window was increased from 0 to 60 s.

Lowering the minimum confidence from 60% to 10%, while keeping the sliding window at 0 s and the minimum relative support at 45%, is presented in Figure 6a,d. More sensors appeared in the consequent part of rules, and the complexity of rules did not change when the minimum confidence was lowered. The low confidence rules imposed high number of false positives for its sensors, which has deteriorated the performance of the system. The false positives induced when the minimum confidence was decreased to 10% (average false positives of 84,178) are much greater than those induced when the minimum relative support was lowered to 2% (average false positives of 29,493). This is because some of the extracted low confidence rules have high support, thus their sensors will be triggered a lot by the user.

#### 4.3.2. Setting Parameters

We aim to select the best combination of values for the sliding window size, minimum relative support, minimum confidence and health threshold, which would enable failure detection and isolation of as many sensors as possible with high precision and recall. The thresholds setting dataset is used to validate the selection. A set of guidelines that aids in the parameters selection process was formulated and is presented as follows:

1. First, extract the association rules for various combinations of values from wide range of sliding window size, minimum relative support and confidence > = 50%, while maintaining a single preliminary threshold value, using the training dataset.
2. Then, sort the combinations of parameters according to the total number of sensors in consequent part of their extracted rules in descending order.
3. Select the top-most set of parameters, which produces rules with the highest number of consequent sensors, then prune this set of rules as illustrated in Section 3.1.3.
4. Use the pruned rules to detect failure when each of the consequent sensors is injected with fail-stop failure in the thresholds setting dataset. Afterwards, plot the all-in-one ROC curve of failure detection, that is plotted with aggregating all the sensor failure cases. Furthermore, plot the individual ROC curves of failure detection when each sensor has failed to have more insights about the performance.
5. Find the optimal operating point and the AUC of the all-in-one ROC curve.
6. If the all-in-one ROC curve shows poor performance, i.e., optimal TPR is low (<0.8), optimal FPR is high (>0.02) and AUC is low (<0.9), then delete this set of parameters entry from the sorted combinations and repeat Steps 3–6 with the next highest number of consequent sensors. Otherwise, the selection process of parameters is done successfully, recording the corresponding sliding window size, minimum relative support and minimum confidence.
7. Record the health threshold value that corresponds to the optimal operating point of the all-in-one ROC curve.

The exclusion of the values of confidence that are below 50% in Step 1 is necessary, as when we experimented with below 50% confidence, its ROC curves had always showed poor performance with optimal TPR below 0.8 and/or optimal FPR above 0.02 and/or AUC below 0.9. In addition, the logic in Algorithm A2 which our calculations for failure detection rely upon in the case of rule satisfaction is sustained while using > = 50% confidence. If we used a low confidence rule, e.g., 10%, and it is satisfied then the probability that the sensors of the satisfied rule are faulty would be 90%, which would make rule satisfaction useless to confirm that its sensors are nonfaulty due to fulfilling the correlation.

**Figure 7:**
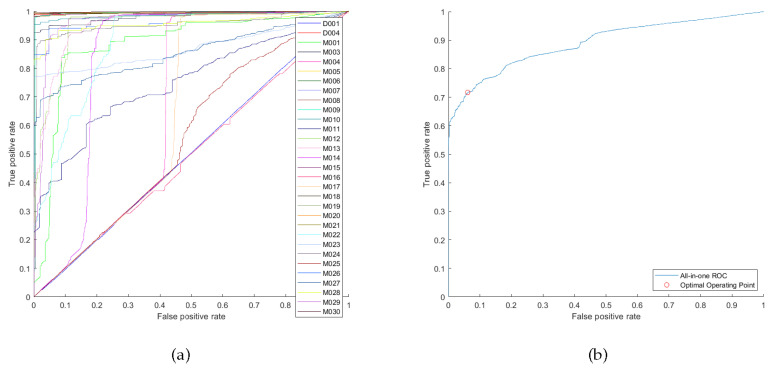
Using sliding window size of 60 s, minimum relative support 5% and minimum confidence of 50%: (**a**) ROC curves of failure detection when each consequent sensor has fail-stop failure. (**b**) All-in-one ROC curve.

**Figure 8:**
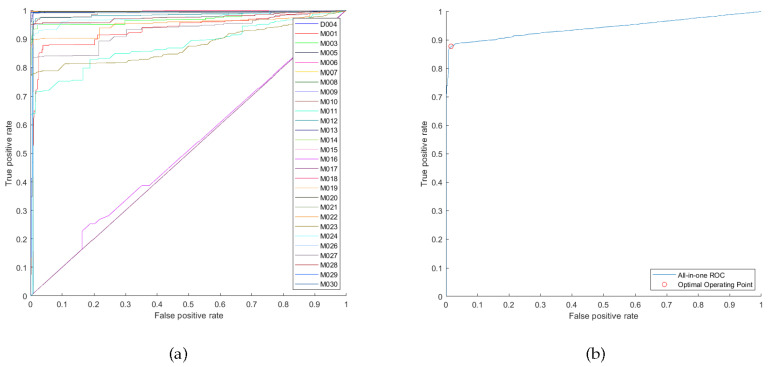
Using sliding window size of 30 s, minimum relative support 15% and minimum confidence of 60%: (**a**) ROC curves of failure detection when each consequent sensor has fail-stop failure. (**b**) All-in-one ROC curve.

To select the parameters for our case study, the proposed guidelines were followed. In Step 1, the set of values we used for the sliding window size was [0, 3, 5, 8, 10, 15, 20, 25, 30, 45, 60] s, the minimum relative support set was [2, 5, 10, 15, 20, 25, 30, 35, 45] %, and the minimum confidence set was [50, 60, 70, 80, 90, 100] %. Note that the number of sensor events of the dataset can be divided by its collection duration to get an estimate about the rate of sensors triggering and accordingly choose the range of set values of the sliding window size. The preliminary health threshold value was chosen to be 0.4. The highest number of consequent sensors that could be obtained using the various combinations of the sets was 31 sensors. However, the values of the parameters that yield 31 consequent sensors produce bad failure detection performance that is reflected on its ROC curves. Figure 7 shows the ROC curves that were plotted from setting the sliding window size to 60 s, minimum relative support to 5% and minimum confidence to 50%, this setting yields rules with 31 consequent sensors. The all-in-one ROC curve has an optimal TPR of 0.7169, optimal FPR of 0.06104 and AUC of 0.8903. Iterating back between Steps 3–6, until good ROC curves in Figure 8 are reached from setting the sliding window size to 30 s, minimum relative support to 15% and minimum confidence to 60%. These finally selected values of parameters could detect failures for 28 sensors. Its all-in-one ROC curve has an optimal TPR of 0.8773, optimal FPR of 0.01593 and AUC of 0.9419. The health threshold value that corresponds to the optimal operating point is 0.3591. Note that it may happen that multiple combinations of parameters for the same number of consequent sensors would produce similar overall performance but with one sensor performing better than the other, and vice versa. In our case study, the previously mentioned selected values for parameters produced close performance to that of using sliding window of 45 s, minimum relative support of 20% and minimum confidence of 60%. However, we favoured our selection because less computational effort during the monitoring stage is needed for the smaller sliding window size.

### 4.4. Experiments

Three types of failures were injected in the testing dataset; fail-stop, obstructed-view and moved-location failures. Each consequent sensor was injected with failure, and the failure detection as well as isolation was evaluated. The initial values of all health status of sensors were set to 1. The sliding window size, minimum relative support, minimum confidence and health threshold were set to 30 s, 15%, 60% and 0.3591, respectively, according to the selection of parameters conducted in Section 4.3.2. The following sensors, D001, D002, M002, M004, M025 and M031, were not checked for failure, as they did not appear in the consequent part of any rule.

#### 4.4.1. Fail-Stop Failure

**Figure 9:**
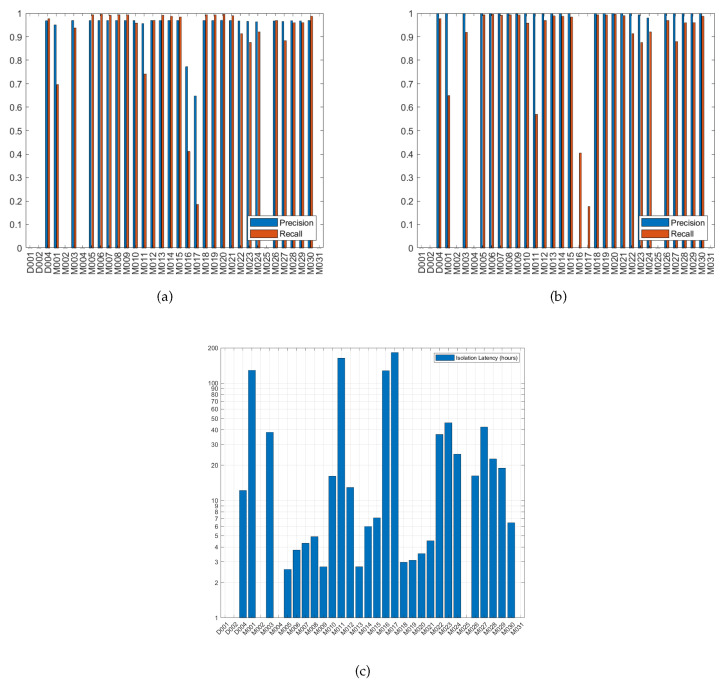
Fail-Stop Failure: (**a**) Precision and recall of failure detection. (**b**) Precision and recall of failure isolation. (**c**) Failure isolation latency.

Fail-stop failure was injected by replacing the readings of the sensor under test by zeros after its point of failure. Fail-stop failure was injected individually on each of the sensors that appeared in the consequent part of rules. The precision and recall of detecting fail-stop failure when failure is injected in each of those sensors is shown in Figure 9a. Meanwhile, the precision and recall for isolating the faulty sensor is shown in Figure 9b. The precision and recall metrics were computed as described in Section 4.2. On the x-axis of Figure 9 lie the IDs of all the event-driven sensors of the apartment shown in Figure 5. The figures are interpreted as follows, the bar columns at sensor D004 in Figure 9a are the precision and recall values of detecting that a failure has occurred when D004 was injected with fail-stop failure. While in Figure 9b, the columns at D004 show the precision and recall of identifying that D004 has failed. No columns were plotted at D001, D002, M002, M004, M025 and M031, as those sensors were not injected with failure nor evaluated as they did not appear as a consequent in the rules. Most of the consequent sensors have high precision and recall for its detection and isolation. There are 26 sensors that when injected with fail-stop failure cause failure detection precision ≥ 0.95, and 24 sensors that cause a recall ≥ 0.87. Isolation precision is ≥ 0.97 for 26 sensors, while the isolation recall is ≥ 0.87 for 24 sensors. The isolation latency was plotted in Figure 9c. The isolation latency is between 2 and 7 h in 13 sensors, 12 and 24 h in 6 sensors and 24 and 48 h in 5 sensors. There are 4 sensors (M001, M011, M016 and M017) that reported very high isolation latency ≥ 120 h. The higher the rate at which the sensor is triggered by the user, i.e., higher support, the shorter the time needed for isolation. It is observed that the sensors which have high isolation precision but along with low isolation recall and high latency, e.g., M001 and M011, are those governed by rules of low support. D002 appears as an antecedent in all the governing rules of M016 and M017, e.g., D002, M019 -> M016. In the first two segments of the testing data, D002 did not have any triggers. Thus, the rules that have M016 and M017 as consequent were never initiated in the first two segments. As a result, M016 and M017 have undefined isolation precision in Figure 9b because of the zero true positives of those two segments. Those segments that have undefined isolation precision were excluded when calculating the average isolation latency for each sensor plotted in Figure 9c. M016 and M017 have high trigger rates but their rules have low support, because one of its antecedent sensors, D002, has a low trigger rate. To calculate the average precision and recall of failure detection and isolation among the examined sensors of the experiment, the two segments of M016 and M017 that had undefined isolation precision were excluded. The average precision and recall of failure detection are 0.9493 and 0.9018, respectively, while the average failure isolation precision and recall are 0.9987 and 0.9116, respectively.

#### 4.4.2. Obstructed-View Failure

**Figure 10:**
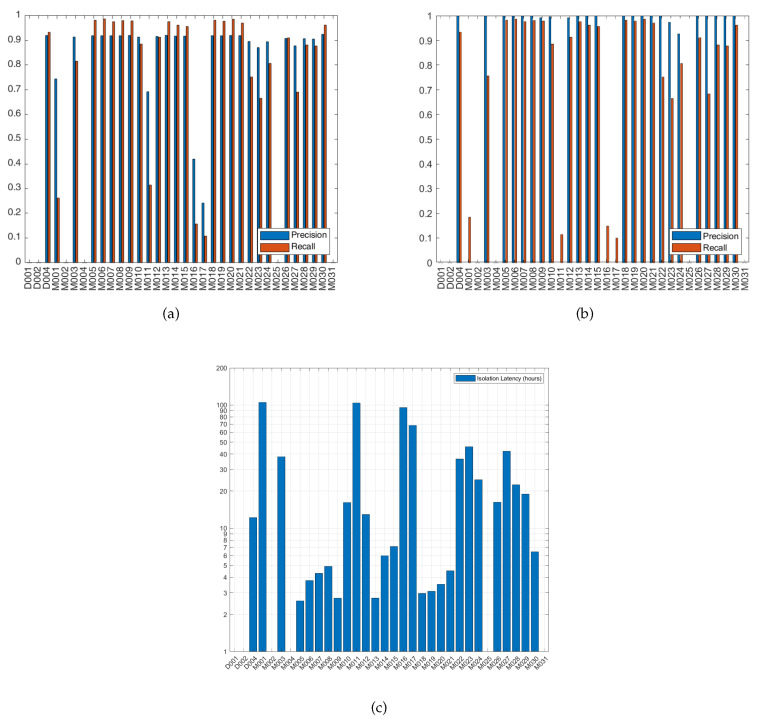
Obstructed-View (5 days) Failure: (**a**) Precision and recall of failure detection. (**b**) Precision and recall of failure isolation. (**c**) Failure isolation latency.

Obstructed-view failure is the failure at which the sensor view is obstructed, e.g., its view gets blocked by furniture. It was simulated by replacing the sensor readings by zeros along the duration at which the sensor view was obstructed. The obstruction duration was set to 5 days. Figure 10a shows the precision and recall of detecting 5 days of obstructed-view failure. The precision and recall for isolating the faulty sensor and its isolation latency are shown in Figure 10b,c, respectively. Similar to the fail-stop failures, detecting and isolating most consequent sensors show high detection and isolation performance except for M001, M011, M016 and M017. There are 20 sensors that when injected with obstructed-view failure cause failure detection precision ≥ 0.9, and 4 sensors between 0.8 and 0.9. Meanwhile, 24 sensors can be isolated with precision ≥ 0.92, and 19 sensors can be isolated with recall ≥ 0.87. The average failure detection precision and recall among examined sensors are 0.8563 and 0.8089, respectively. The average failure isolation precision and recall are 0.9954 and 0.8285, respectively. The isolation latency for the sensors injected with obstructed-view failure is almost the same as when injected with fail-stop failure.

#### 4.4.3. Moved-Location Failure

**Figure 11:**
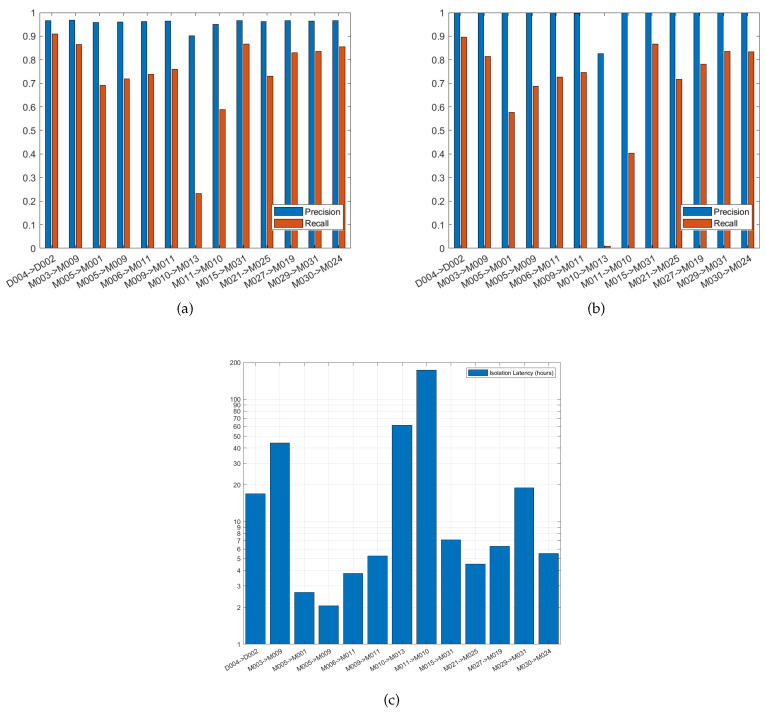
Moved-Location Failure: (**a**) Precision and recall of failure detection. (**b**) Precision and recall of failure isolation. (**c**) Failure isolation latency.

Moved-location failure means that a sensor’s location has changed, this may happen when a sensor gets remounted by the user in the wrong location or when it is mounted on a piece of furniture that has been moved to another location. This type of failure was simulated by changing the readings of the sensor after its point of failure by readings of its newly moved location. Figure 11 shows the performance of detecting and isolating the moved-location of some of the consequent sensors. The x-axis of Figure 11 describes the moved-location case, e.g., D004 -> D002, means that the sensor D004 has moved to the location of sensor D002. Figure 11a plots the precision and recall of detecting failure, and Figure 11b shows the precision and recall of identifying that the moved sensor has failed, i.e., the failed sensor is D004 in our previous example. The precision of failure detection in the presented 13 moved-location cases are ≥0.9, and the precision of the failure isolation is ≥0.99 in the presented cases except for M010 -> M013 is 0.83. On the other hand, the recall of failure detection is ≥0.82 for 6 cases, between 0.7 and 0.8 for 5 cases, and ≤ 0.6 for 2 cases. Meanwhile, the recall of failure isolation is ≥0.8 for 5 cases, between 0.68 and 0.8 for 5 cases, and ≤0.6 for 3 cases. The average failure detection precision and recall among the presented cases are 0.9580 and 0.74, respectively, while the average failure isolation precision and recall are 0.9863 and 0.6839, respectively. The isolation latency is ≤7 h in 8 cases, between 16 and 19 h in 2 cases, and ≥42 h in 3 cases. The distance of the new location from the old one is not what dominates the precision or recall of detecting the moved-location failure. Moving a sensor within the same room could be detected with higher recall when M005 was moved to the location of M001 within the bedroom than that of moving M010 to M013 within the living room. Similarly, moving a sensor to another room could be detected with higher recall when D004 was moved from the garage door to replace the D002 at the kitchen back door than that of moving M005 from the bedroom to M009 in living room.

## 5. Discussion

Our proposed failure detection and isolation system is distinguished by its low computational effort and high interpretability, in addition to its use of unlabelled datasets. The results show that the consequent sensors that were injected with fail-stop and obstructed-view failures could be detected and isolated with high precision and recall. The isolation latency is highly dependable on the behaviour of the resident as well as the start time of failure with respect to his behaviour. The more frequent the usage of the area of an apartment that has the failed sensor is, the shorter the time to isolate this sensor failure. In addition, the start time of the failure affects the isolation latency, i.e., if the sensor failure has occurred just before the resident goes to bed at night, then the failure will not be isolated before the next morning by any means. Detecting moving a sensor to another place can be achieved with high precision and recall only when this newly moved location has minimal correlation to the old location. This is on contrary to the fail-stop and obstructed-view failures, where the sensor failure detection performance is proportional to its correlation to other sensors.

A summary table of the related work was presented in our survey paper [11]. Although the results are not directly comparable due to the use of different datasets, design of experiments and evaluation methodology, the benefits of our proposed system over the other relevant state of the art was presented in Section 2. The limitation of our approach is that the sensors that do not appear as consequent to the activation of other sensor(s) in the apartment cannot be checked for failure. However, our approach can be used to determine these sensors, and thus can help to highlight the needed reconfiguration of sensors’ positioning in the apartment to obtain a fully functional sensor failure detection and isolation system.

As for future work, the use of variable size sliding window for detecting failures may further improve the system performance, especially for the moved-location failures. Rules will be extracted for the consequent sensors that have strong rules using shorter duration sliding window during the correlations extraction stage, and only those sensors that did not appear will be extracted over a longer duration sliding window. However, this should be weighed against its computational complexity during the real-time correlations monitoring stage. Furthermore, the use of an auxiliary system to detect failure for those sensors that did not appear as consequent could be investigated. This auxiliary system may exploit the following features for those sensors; its trigger day, trigger time and duration of activation.

## 6. Conclusions

This paper proposed a failure detection and isolation system for binary event-driven sensors deployed in the AAL environment. Correlations between sensors were extracted with no prior knowledge of the sensor placement on the floor plan and using unlabelled datasets. Guidelines for the selection of the user defined parameters for correlations extraction were presented. The correlations are monitored during run-time to detect sensor failures. The proposed approach was evaluated using publicly available dataset injected with fail-stop, obstructed-view and moved-location failures. The system was able to detect and isolate the various types of failures. The results show that fail-stop failures could be detected with an average precision and recall of 0.9493 and 0.9018, and isolated with average precision and recall of 0.9987 and 0.9116, respectively. Obstructed-view failures were detected with average precision of 0.8563 and recall of 0.8089, and isolated with average precision of 0.9954 and recall of 0.8285. Meanwhile, the moved-location failures were detected at 0.9580 average precision and at 0.74 average recall and isolated at 0.9863 average precision and 0.6839 average recall.

## Abbreviations

The following abbreviations are used in this manuscript:

AAL	Ambient assisted living
ICT	Information and communication technologies
AmI	Ambient intelligence
ADL	Activities of daily living
PIR	Passive infrared sensor
TP	True positives
FN	False negatives
FP	False positives
TN	True negatives
ROC	Receiver operating characteristic
AUC	Area under curve
TPR	True positive rate
FPR	False positive rate

## Appendix A

**Algorithm A1** Failure detection.
**Input:**
*DataStream*: the stream of the AAL sensors *events*
*Sen*: the set of sensors represented by tuples {(id, Health, FailFlag)}, where id is the sensor’s id
number, Health is the health status of sensor, and FailFlag is the failure flag of sensor
*R*: the set of rules represented by tuples {(Ant, Conseq, Sup, Conf)}, where Ant contains the sensors
in the rule antescedent, Conseq contains the sensors in the rule consequent, Sup is the support of
rule, and Conf is the rule’s confidence
SatRulHist: the set of rules that were satisfied in the previous sliding window SwNum−1, where
SwNum is the sliding window’s running number
UnSatRulHist: the set of rules that were unsatisfied, i.e., has one missing sensor in the rule
consequent, in the previous sliding window SwNum−1
FutSw: the set of sensors that are active in the next sliding window SwNum+1
HealthThresh: the threshold value for the health status of sensors
**Output:**
Sen: updated Health and FailFlag of the set of sensors
1: **while** CurrSW= ProcessSW(DataStream) **do**
2: // CurrSw is the set of active sensors in the current sliding window SwNum
3: **for each** Rul∈R∧Rul.Ant⊆CurrSw **do**
4: **if** Rul.Conseq⊂CurrSw **then**
5: Sen.Health← SatisfHealthUpdate(Rul, CurrSw, Sen)
6: SatRulHist←Rul
7: **else if** |Rul.Conseq−CurrSw|=1 **then**
8: **if** Rul∉SatRulHist∧|(Rul.Ant∪Rul.Conseq)−FutSw|≠ϕ **then**
9: **if** Rul∉UnSatRulHist **then**
10: Sen.Health← UnSatisfHealthUpdate(Rul,CurrSw,Sen)
11: **end if**
12: UnSatRulHist←Rul
13: **end if**
14: **end if**
15: **end for**
16: **for each** s∈Sen **do**
17: **if** s.Health<HealthThresh **then**
18: s.FailFlag←1
19: **else**
20: s.FailFlag←0
21: **end if**
22: **end for**
23: **end while**

**Algorithm A2** Health Status Update due to Rule Satisfaction.
1: **SatisfHealthUpdate** {Rul, CurrSw, Sen} 2: **for each**s∈Sen, s.id∈ (Rul.Ant∪Rul.Conseq) **do** 3: PrF←1−Rul.Conf // PrF is the probability that the sensor is faulty 4: s.Health←0.1×(1−PrF)+0.9×s.Health 5: **end for** 6: **return** Sen.Health

**Algorithm A3** Health Status Update due to Rule UnSatisfaction.
1: **UnSatisfHealthUpdate** {Rul,CurrSw,Sen} 2: **for each**s∈Sen, s.id∈Rul.Ant**do** 3: **if** |Rul.Conseq|=1∧|Rul.Ant|=1 **then** 4: PrF←1−P(s) 5: **else** 6: $PrF\leftarrow 1-\frac{P({⋂}_{\left\{x\in (CurrSW\cap (Rul.Ant\cup Rul.Conseq)\right\}}x)}{P({⋂}_{\left\{x\in (CurrSW\cap (Rul.Ant\cup Rul.Conseq))|x\neq s\right\}}x)}$ 7: **end if** 8: s.Health←0.1×(1−PrF)+0.9×s.Health 9: **end for** 10: **for each**s∈Sen, s.id∈Rul.Conseq**do** 11: **if** s.id∈CurrSW **then** 12: $PrF\leftarrow 1+\frac{Rul.Sup-P({⋂}_{\left\{x\in (CurrSW\cap (Rul.Ant\cup Rul.Conseq))\right\}}x)}{P\left({⋂}_{\left\{x\in (CurrSW\cap (Rul.Ant\cup Rul.Conseq))|x\neq s\right\}}x\right)-P\left({⋂}_{\left\{x\in (Rul.Ant\cup Rul.Conseq)|x\neq s\right\}}x\right)}$ 13: **else** 14: $PrF\leftarrow \frac{Rul.Sup}{P({⋂}_{\left\{x\in (CurrSW\cap (Rul.Ant\cup Rul.Conseq))\right\}}x)}$ 15: **end if** 16: s.Health←0.1×(1−PrF)+0.9×s.Health 17: **end for** 18: **return** Sen.Health

## References

[1] Nations U. (2019). World Population Ageing.

[2] Rashidi P., Mihailidis A. (2013). A Survey on Ambient-Assisted Living Tools for Older Adults. IEEE J. Biomed. Health Inform..

[3] Monekosso D., Florez-Revuelta F., Remagnino P. (2015). Ambient Assisted Living [Guest editors’ introduction]. IEEE Intell. Syst..

[4] Dobre C., Mavromoustakis C.X., Garcia N.M., Mastorakis G., Goleva R.I. (2017). Introduction to the AAL and ELE Systems. Ambient Assisted Living and Enhanced Living Environments.

[5] Viard K., Fanti M.P., Faraut G., Lesage J.J. An event-based approach for discovering activities of daily living by hidden Markov models. Proceedings of the 2016 15th International Conference on Ubiquitous Computing and Communications and 2016 International Symposium on Cyberspace and Security (IUCC-CSS).

[6] Kapitanova K., Hoque E., Stankovic J.A., Whitehouse K., Son S.H. Being SMART about failures: Assessing repairs in SMART homes. Proceedings of the 2012 ACM Conference on Ubiquitous Computing.

[7] Rahal Y., Pigot H., Mabilleau P. (2008). Location Estimation in a Smart Home: System Implementation and Evaluation Using Experimental Data. Int. J. Telemed. Appl..

[8] Feng Z., Fu J.Q., Wang Y. Weighted distributed fault detection for wireless sensor networks Based on the distance. Proceedings of the 33rd Chinese Control Conference.

[9] Fan C., Tan J. A majority voting scheme in wireless sensor networks for detecting suspicious node. Proceedings of the 2009 Second International Symposium on Electronic Commerce and Security.

[10] Nguyen T.A., Bucur D., Aiello M., Tei K. Applying time series analysis and neighbourhood voting in a decentralised approach for fault detection and classification in WSNs. Proceedings of the Fourth Symposium on Information and Communication Technology.

[11] ElHady N.E., Provost J. (2018). A Systematic Survey on Sensor Failure Detection and Fault-Tolerance in Ambient Assisted Living. Sensors.

[12] Ballardini A.L., Ferretti L., Fontana S., Furlan A., Sorrenti D.G. (2016). An indoor localization system for telehomecare applications. IEEE Trans. Syst. Man Cybern. Syst..

[13] Ahvar E., Lee G.M., Han S.N., Crespi N., Khan I. (2016). Sensor network-based and user-friendly user location discovery for future smart homes. Sensors.

[14] Mckeever S., Ye J., Coyle L., Bleakley C., Dobson S. (2010). Activity recognition using temporal evidence theory. J. Ambient. Intell. Smart Environ..

[15] Javadi E., Moshiri B., Yazdi H.S. (2013). Activity Recognition In Smart Home Using Weighted Dempster-Shafer Theory. Int. J. Smart Home.

[16] Amri M.H., Aubry D., Becis Y., Ramdani N. (2015). Robust fault detection and isolation applied to indoor localization. IFAC-PapersOnLine.

[17] Danancher M. (2013). A Discrete Event Approach for Model-Based Location Tracking of Inhabitants in Smart Homes. Ph.D. Thesis.

[18] Veronese F., Pour D.S., Comai S., Matteucci M., Salice F. (2014). Method, Design and Implementation of a Self-checking Indoor Localization System. International Workshop on Ambient Assisted Living.

[19] Veronese F., Comai S., Matteucci M., Salice F. Method, design and implementation of a multiuser indoor localization system with concurrent fault detection. Proceedings of the 11th International Conference on Mobile and Ubiquitous Systems: Computing, Networking and Services, ICST (Institute for Computer Sciences, Social-Informatics and Telecommunications Engineering).

[20] Mohamed A., Jacquet C., Bellik Y. A fault detection and diagnosis framework for ambient intelligent systems. Proceedings of the Ubiquitous Intelligence & Computing and 9th International Conference on Autonomic & Trusted Computing (UIC/ATC).

[21] Jacquet C., Mohamed A., Bellik Y. (2013). An ambient assisted living framework with automatic self-diagnosis. Int. J. Adv. Life Sci..

[22] Oliveira C.H.S., Giroux S., Ngankam H., Pigot H. Generating Bayesian Network Structures for Self-diagnosis of Sensor Networks in the Context of Ambient Assisted Living for Aging Well. Proceedings of the International Conference on Smart Homes and Health Telematics.

[23] Munir S., Stankovic J.A. Failuresense: Detecting sensor failure using electrical appliances in the home. Proceedings of the Mobile Ad Hoc and Sensor Systems (MASS).

[24] Kodeswaran P.A., Kokku R., Sen S., Srivatsa M. Idea: A system for efficient failure management in smart iot environments. Proceedings of the 14th Annual International Conference on Mobile Systems, Applications, and Services.

[25] Ye J., Stevenson G., Dobson S. Using temporal correlation and time series to detect missing activity-driven sensor events. Proceedings of the Pervasive Computing and Communication Workshops (PerCom Workshops).

[26] Ye J., Stevenson G., Dobson S. Fault detection for binary sensors in smart home environments. Proceedings of the Pervasive Computing and Communications (PerCom).

[27] Ye J., Stevenson G., Dobson S. (2016). Detecting abnormal events on binary sensors in smart home environments. Pervasive Mob. Comput..

[28] Kapitanova K., Hoque E., Stankovic J.A., Son S.H., Whitehouse K., Alessandrelli D. (2011). Being SMART About Failures: Assessing Repairs in Activity Detection. https://www.semanticscholar.org/paper/Being-SMART-About-Failures-%3A-Assessing-Repairs-in-Kapitanova-Hoque/f05968403b88738e869a360ca3910bebad5218b4#citing-papers.

[29] Choi J., Jeoung H., Kim J., Ko Y., Jung W., Kim H., Kim J. Detecting and identifying faulty IoT devices in smart home with context extraction. Proceedings of the 2018 48th Annual IEEE/IFIP International Conference on Dependable Systems and Networks (DSN).

[30] Agrawal R., Imieliński T., Swami A. (1993). Mining Association Rules between Sets of Items in Large Databases.

[31] Yairi T., Kato Y., Hori K. Fault detection by mining association rules from house-keeping data. Proceedings of the International Symposium on Artificial Intelligence, Robotics and Automation in Space.

[32] Hou Z., Lian Z., Yao Y., Yuan X. (2006). Data mining based sensor fault diagnosis and validation for building air conditioning system. Energy Convers. Manag..

[33] Liu J., Shi D., Li G., Xie Y., Li K., Liu B., Ru Z. (2020). Data-driven and association rule mining-based fault diagnosis and action mechanism analysis for building chillers. Energy Build..

[34] Agarwal R., Srikant R. Fast algorithms for mining association rules. Proceedings of the 20th VLDB Conference.

[35] Jacquenet F., Largeron C., Udréa C. Efficient management of non redundant rules in large pattern bases: Bitmap approach. Proceedings of the Eighth International Conference on Enterprise Information Systems: Databases and Information Systems Integration.

[36] CASAS Datasets. http://ailab.wsu.edu/casas/datasets/.

